# Enhancing mental health care: a problem, resources and goals oriented multidimensional framework (PRoGO)

**DOI:** 10.1007/s00406-025-02045-5

**Published:** 2025-07-01

**Authors:** Stefan Leucht, Bernhard König, Antonio di Francesco, Alessandro Rodolico, Josef Priller, Kerem Böge, Wolfgang Strube, Jochen Gensichen, Peter Bechmann, Amr ElDeeb, Alkomiet Hasan, Steffen Moritz, Markus Jäger, Claudia Leucht, Jim van Os, John M. Davis

**Affiliations:** 1 Department of Psychiatry and Psychotherapy, Klinikum Rechts Der Isar, Technical University of Munich, TUM School of Medicine and Health, Ismaningerstrasse 22, 81675 Munich, Germany; 2 German Center for Mental Health (Partner Site Munich-Augsburg), Munich, Germany; 3 Praxis Deisenhofen, Jägerstr. 16a, 82041 Oberhaching, Germany; 4 Department of Clinical and Experimental Medicine, Institute of Psychiatry, University of Catania, Catania, Italy; 5 Neuropsychiatry, Charité—Universitätsmedizin Berlin and Deutsches Zentrum Für Neurodegenerative Störungen, 10117 Berlin, Germany; 6 University of Edinburgh and UK DRI, Edinburgh, UK; 7 Charité Universitätsmedizin Berlin, Berlin, Germany; 8 German Center for Mental Health (Partner Site Berlin), Berlin, Germany; 9 Medizinische Hochschule Brandenburg, Neuruppin, Germany; 10 Department of Psychiatry, Psychotherapy and Psychosomatics, Medical Faculty, University of Augsburg, Augsburg, Germany; 11 Institute of General Practice and Family Medicine, University Hospital, LMU Munich, Munich, Germany; 12 KBO-Inn-Salzach Klinikum, Wasserburg Am Inn, Germany; 13 Department of Psychiatry and Psychotherapy, University Medical Center Hamburg-Eppendorf, Hamburg, Germany; 14 District Hospital Kempten, Kempten, Germany; 15 Max Planck Institute of Psychiatry, Munich, Germany; 16 Department of Psychiatry, UMC Utrecht Brain Center, University Medical Center Utrecht, Utrecht University, Utrecht, The Netherlands; 17 Department of Psychosis Studies‚ Institute of Psychiatry‚ Psychology and Neuroscience‚ King’s College, London, United Kingdom; 18 King’s College London, London, UK; 19 Psychiatric Institute, University of Illinois at Chicago, Chicago, IL USA; 20 Johns Hopkins University, Baltimore, MD USA

**Keywords:** Problem-oriented medical records, Transdiagnostic, Lawrence Weed, Psychiatry, Mental health, Schizophrenia, Depression, Anxiety

## Abstract

**Background:**

Lawrence Weed introduced the “Problem-Oriented Medical Records” (POMR) approach to medicine. Its core principle is that treatment should be organised around patients’ specific problems. This approach gained widespread adoption in the United States during the 1970s. However, few studies have compared POMR with the traditional “source-based” method, and evidence supporting its application in mental health remains particularly limited.

**Methods:**

We carried out a narrative review to examine whether POMR is appropriate for mental health care and which modifications are necessary for this purpose.

**Findings:**

Psychiatry and psychotherapy address brain-mediated disorders that lack clear biological markers. Diagnoses rely on the assessment of psychopathological symptoms, which are grouped into syndromes. These symptoms and syndromes can be effectively categorized as “problems”. Given that psychiatric treatment is often symptomatic in that it focuses on symptoms rather than diagnoses, POMR provides an ideal framework for organizing care.

Furthermore, mental health is inherently multidimensional, encompassing biological, psychological, social, and existential domains. This complexity necessitates interdisciplinary collaboration. However, under the conventional source-based approach, professional groups often operate in parallel rather than jointly together. POMR, by contrast, facilitates seamless collaboration by aligning teams around patient-centred problems. A special aspect of mental health care is the emphasis on considering patients’ individual goals and resources, rather than focusing only on their deficits.

**Interpretation:**

By systematically applying POMR to psychiatric care—particularly through tightly coordinated interdisciplinary treatment—clinicians could enhance both clinical and functional outcomes, improve both patient and team satisfaction, and better align treatment with patients’ unique needs. To support this approach, we propose a practical grid which we refer to as the Problem–Resources–Goals Oriented framework (PRoGO), reflecting the necessary adaptations for mental health. Clinical trials are warranted to assess the effectiveness of POMR/PRoGO in psychiatric practice and its potential to advance the field.

**Supplementary Information:**

The online version contains supplementary material available at 10.1007/s00406-025-02045-5.

## Introduction

Mental health is a complex field encompassing biology, psychology, sociology and even philosophy. Most psychiatric disorders are currently understood as an interplay of these various dimensions [1, 2]. For instance, the diathesis-stress model suggests that individuals with biological vulnerabilities may develop psychiatric disorders when exposed to stressors such as emotional trauma [3].

Moreover, mental-health problems often manifest as an overlap of various psychopathological dimensions. Examples include schizoaffective disorder, which exists on a continuum between schizophrenia and affective disorders; depression and anxiety, which frequently co-occur; and the phenomenological overlap between bipolar disorder and ADHD.

The diagnostic systems DSM and ICD do not fully capture this dimensionality. They are predominantly categorical, driven in part by administrative and re-imbursement considerations. Moreover, as there are no definitive biomarkers DSM/ICD diagnostic criteria are ultimately determined by consensus. Dimensional diagnostic approaches such as the Hierarchical Taxonomy of Psychopathology (HiTOP) [4], Research Domain Criteria (RDoC), network [5–7] and staging [8] approaches are under development. However, all have limitations so that some have proposed to focus on psychopathological signs and symptoms which are nearer to nature than consensus-based DSM/ICD symptoms [9].

These interplays make mental health more complex than most other medical specialties, where diagnoses and treatment are often more straightforward.

One major practical implication of mental health’s multi-dimensionality is that clinicians must address not only the psychiatric disorder itself but also the broader issues stemming from other dimensions. For instance, unemployment or family problems may contribute to and perpetuate a depressive episode, and addressing these issues is integral to effective treatment.

Traditionally, medical records are organized in a source-oriented manner, grouping information based on its origin, such as laboratory reports, radiology results, or physician and nurse notes. This system emphasizes the *source* of information rather than the specific *problems* it addresses. In the 1960 s, Lawrence Weed proposed a shift to a problem-oriented structure, where all relevant data related to a specific patient issue are organized together.

This “problem-oriented medical record” (POMR) system was designed to enhance the clarity and accessibility of patient information, facilitate collaboration among healthcare providers, reduce data duplication, and improve the ability to identify patterns and connections in a patient’s medical history [10]. POMR has been widely adopted in medicine. Curiously, however, despite psychiatric care meaning problem-solving across multiple domains, POMR has not been extensively applied in the psychiatric field.

In this review we work out how integrating problem oriented medical records (POMR) with psychiatry specific elements such as goal- and resource orientation, and a focus on psychopathological phenomena and individual characterization across biological, psychological and social dimensions rather than diagnoses—could enhance psychiatric care. It has the potential to improve treatment efficacy, patient satisfaction, team satisfaction, teaching, and ultimately, patient outcomes. We refer to this approach as the Problem–Resources–Goals Oriented framework (PRoGO), reflecting the necessary adaptations for mental health.

## The problem-oriented medical record system (POMR) by Laurence Weed

**Fig. 1:**
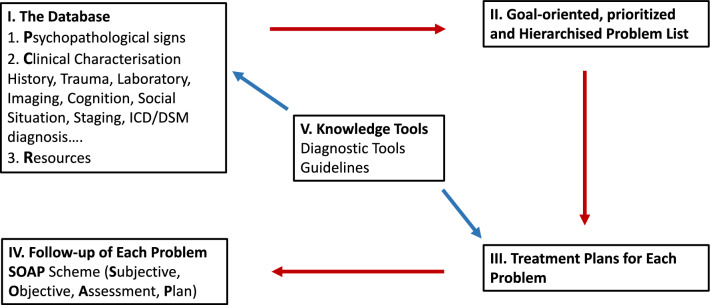
Problem-oriented Medical Records (modified for psychiatry from Weed and Weed 2011 [10])

Laurence Weed argued that the structure of medical data influences how clinicians think and treat patients. He emphasized that the practice of medicine and the organization of medical records are inseparable and inherently intertwined. Moreover, he recognized early on that the human brain cannot retain all the necessary information about each patient, recall all medical knowledge by heart, and integrate both effectively during treatment. To address this limitation, he proposed that medical records should be organised around a patient’s specific problems, and linked to knowledge databases. This approach would ensure that clinicians focus on patients’ needs, enabling more targeted and effective care. The “problem-oriented medical record” (POMR) consists of four key components (see Fig. 1) [11]:

- 1. The database

The database encompasses all relevant information about the patient to ensure a comprehensive characterization. During this process, clinicians must systematically gather information by asking targeted questions to document both the presence of symptoms (pertinent positives) and their confirmed absence (pertinent negatives, such as explicitly noting the absence of hallucinations).

- 2. The problem list

This is a hierarchical list of all the patient’s current relevant problems which is reached after a brainstorming phase between team members. It tends to be more comprehensive than a mere list of diagnoses, because only problems that are understood at the level of a diagnosis would make it onto the latter. It provides a clear overview of a patient’s issues at a glance, eliminating the need to shift through the entire chart and ensuring no problems are overlooked. The hierarchical aspect helps prioritizing issues, such as determining which problems can be taken care of during an inpatient stay.

- 3. Treatment plans

For each problem listed, a specific treatment plan is devised.

- 4. Progress notes

They are organised in the SOAP format:

- Subjective (S): The patient’s report of his condition.
- Objective (O): Data from laboratory tests, technical studies, and physical examinations.
- Assessment (A): The clinician’s interpretation of the subjective and objective data.
- Plan (P): The proposed treatment plan based on the assessment.

The last two components bring a crucial advantage over the source-oriented record. By interpreting subjective and objective data in relation to specific problems, clinicians can clearly plan and articulate the next therapeutic steps.

POMR gained widespread acceptance in the USA during the 1970s. According to a 1972 survey, 82% of all medical schools in the USA taught problem-oriented charting [12, 13]. Weed also developed one of the first electronic health record systems, PROMIS (Problem-Oriented Medical Information System) [14]. However, in 2011, Weed criticized in the article “Medicine in Denial” that the implementation of POMR had stagnated [11]. Today numerous electronic health record systems exist, many of which allow for the creation of problem lists. Nevertheless, the main aim of these systems often leans more toward documentation for financial purposes. The emphasis on problem orientation varies considerably across different electronic health record systems [12].

## Scientific evidence for POMR in general

Despite its widespread adoption in practice, few studies have rigorously evaluated the potential benefits of the Problem-Oriented Medical Record (POMR). Chowdry et al. [12] conducted a review of problem-oriented charting by searching PubMed using the term (Medical Records, Problem-Oriented[MeSH Major Topic]) OR problem-oriented charting (877 results as of August 16, 2024). They identified 15 validation studies that examined a range of outcomes across various fields. Some studies reported benefits such as improved documentation, reduced medication cost, enhanced education, increased use of evidence-based treatment, and better management of obesity. However, overall, the results were mixed. In 2011, Weed and Weed [11] reported only two randomized controlled trials comparing the use of information-coupling software to standard care. One trial, focusing on diabetes care, demonstrated substantial benefits [15], while the other, involving a range of diagnoses, showed no significant advantages [16].

## Scientific evidence for POMR in psychiatry

A Pubmed search using the terms (“Lawrence Weed” OR “Problem-oriented” OR POMR OR SOAP OR POC) AND (“Mental Health” OR Psych* OR Neuropsychiatry OR Depress* OR Anxiety OR Bipolar OR Schizo* OR “Personality disorder*” OR “Mood Disorder*” OR “Eating Disorder*” OR “Post-traumatic” OR PTSD OR “Obsessive–compulsive” OR OCD OR Addiction* OR Neuroscience OR “Cognitive-Behavioral” OR “Cognitive Behavioral” OR Antidepressant* OR Antipsychotic* OR “Mood Stabilizer*” OR Suicide OR Mindfulness) (last search 26/09/2024) yielded 1805 hits. Of these, 23 articles were deemed relevant.

With one exception—a survey by Bakel et al. 2014 [17]—all reports were old, published between 1973 and 1985. The articles centred around the themes “Presentation of the Problem-Oriented Approach”, “Technical Aspects of the P-O Approach”, “Evaluation of the Effectiveness of the P-O Approach” and “Interventions to Increase the Application of the P-O Approach”. Notably, no controlled trials were identified. Consequently, no clear evidence regarding the effectiveness of POMR can be drawn (see eAppendix 1).

## POMR and teaching

Proponents of POMR emphasize its ability to enhance clinical reasoning skills. Every patient encounter becomes an exercise in “problem-based learning,” which is a highly effective educational approach. This method is particularly beneficial for medical students and early-career clinicians. By encouraging structured thinking—identifying problems, formulating treatment plans, and continuously re-evaluating them—POMR fosters critical thinking. Indeed, as illustrated in Fig. 1 through Fig. 4, each issue must be carefully analysed when applying POMR to a patient.

## POMR and evidence-based medicine

In his final book, Weed, along with other critics [18], highlighted a limitation of evidence-based medicine (EBM): recommendations for individual patients cannot always be drawn from average results obtained in randomized controlled trials (RCTs) [11]. The issue of generalizability (external validity) of RCT findings, conducted under idealized conditions (internal validity), to routine clinical care is well-recognized. Individualized treatment is also a stated goal of EBM [19]. In practice, deviations from evidence are often necessary. Despite notable exceptions—such as certain areas of cancer treatment—individualized care and precision medicine remain aspirational rather than widespread realities. Thus, for now, there is no viable alternative to integrating EBM within the framework of POMR.

## Application of POMR in psychiatry

POMR is particularly well-suited to mental health care, as problem-solving lies at the heart of this specialty more than any other area of medicine. Psychiatry and psychotherapy are inherently multidimensional and these dimensions can be seen as problems which need to be addressed to improve patients’ mental health [9].

First, mental health diagnoses are dimensional, often existing along a continuum between health and severe illness, making it challenging to draw the line. Significant overlap between disorders is common. For instance, depression and anxiety are prevalent across most mental health conditions, and affective disturbances are part of the description of schizophrenia in the ICD-11 [9].

Many psychiatric disorders are likely mediated by alterations in brain networks rather than clearly localizable structural changes, as seen in conditions like stroke or multiple sclerosis. These network alterations could explain why the same individuals may experience schizophrenic, schizoaffective, and depressive episodes throughout their lives, and why bipolar disorder can present with opposing symptoms during depressive and manic episodes.

Psychiatric diagnoses are also “polythetic,” meaning that multiple, sometimes mutually exclusive, constellations of symptoms can lead to the same diagnosis. Currently, there are no biomarkers available for diagnosing psychiatric disorders. In fact, psychiatric conditions are, to some extent, by definition those brain diseases for which visible pathology is absent. Diagnosis ultimately relies on the assessment of psychopathological signs and syndromes.

Moreover, most psychotropic drugs target specific symptoms rather than the consensus-based diagnoses outlined in the DSM or ICD. For instance, antipsychotics primarily target psychotic symptoms, largely independent of the underlying diagnosis, and some also function as mood stabilizers [20]. Antidepressants are effective not only for treating depression but also for managing anxiety, obsessive–compulsive symptoms, and depression associated with schizophrenia [21]. Relying on ICD/DSM diagnosis-based pharmacotherapy can even lead to suboptimal outcomes. Using antipsychotics for patients with schizophrenia who present solely with negative symptoms is ineffective. Dopamine receptor blockade may worsen negative symptoms and cognitive impairments rather than alleviate them [22]. In other words, psychotropic treatment is largely symptom-focused. This symptom-focus also applies to cognitive-behavioural psychotherapy, where interventions such as exposure or cognitive restructuring, are utilized across a range of psychiatric conditions. Therefore, we argue that clinicians should prioritize psychopathological signs in their assessments and treatments. They are closer to nature than consensus-based and thus questionable ICD/DSM criteria [23].

Second, much more than somatic diseases, psychiatric problems are an interplay of multiple dimensions. Psychology evidently plays a major role, with factors such as childhood or recent traumatic events, conditioning, instrumental learning, and coping styles influencing mental health. There is also a significant social dimension in that factors such as poverty, discrimination, loneliness, unemployment, being physically handicapped, marital status, family support and related factors play a role in the development and sustainment of mental problems. In addition to genetic factors, the use of illicit drugs such as cannabis can be considered a contributing biological factor [3]. Moreover, somatic diseases and medication for them can provoke psychiatric symptoms. All of these dimensions represent problems which need to be addressed making POMR a perfect fit for psychiatric care.

Third, the treatment teams are inherently multidimensional. As in other specialties, there are physicians and nurses, but in psychiatry, psychotherapists, (neuro)-psychologists, occupational therapists, physical therapists, peer-support workers and social workers play a major role. Nevertheless, as long as “source-oriented records” are used, these groups sometimes tend to work independently rather than jointly together.

**Fig. 2:**
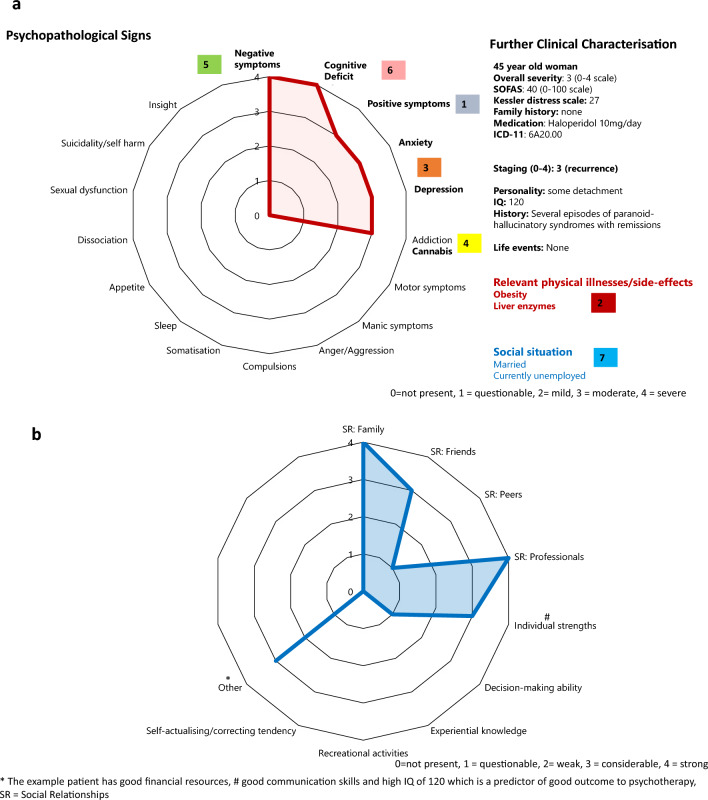
**a** I. The Database—Symptoms and Clinical Characterisation. *SOFAS* Social and Occupational Functioning Assessment Scale [45]. **b** I. The Database—Resources. *The example patient has good financial resources, # good communication skills and high IQ, *SR* Social Relationships. The major categories personal resources according to the review by Priebe et al. [42] are presented. As social relationships (SR) were particularly important, we present the major sub-categories according to Priebe et al. [42]

**Fig. 3:**
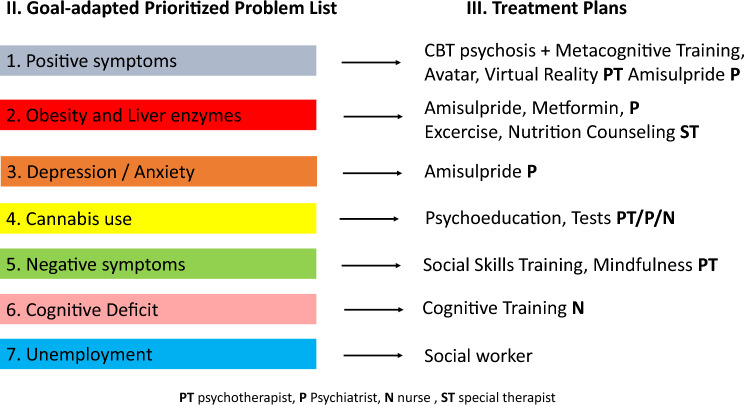
Goal-adapted prioritized problem list and treatment plans

**Fig. 4:**
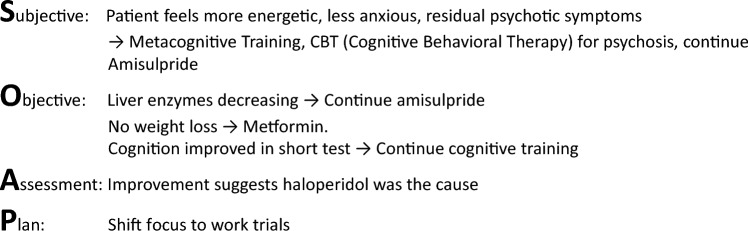
Follow-up (SOAP scheme)

In the following text, we describe how the Problem-Oriented Medical Record (POMR) can be applied to psychiatry and discuss the necessary adaptations, using Weed’s four steps as a framework. Figures 2, 3 and 4 illustrate this approach with an example patient.

- 1. The database

In somatic medicine, the database is built using physical examinations, laboratory tests, imaging, and other diagnostic methods. In mental health care, these diagnostics are primarily employed to rule out rare organic causes, serving as crucial “pertinent negatives” that should be documented.

In mental health care, the “database” consists of *1. psychopathological signs, 2. contextual characterization and 3. resources*, see Figs. 2a and b.

Psychopathological Signs (Fig. 2a, left): Patients undergo an initial *psychopathological assessment* using a cross-disorder instrument. The resulting psychopathological signs are visualized in a spider-net plot, enabling the clinician to quickly identify the patient’s key psychopathological issues that require attention. While an ICD or DSM diagnosis is made for purposes such as communication and billing, it is not the central focus of this process.

The currently probably best transdiagnostic rating scale is the Association-for-Methodology-and-Documentation-in-Psychiatry (AMDP) system [24–26]. This scale contains 100 operationalized and manualized mental symptoms, along with 40 somatic symptoms. They are rated following an interview guide as not present, mild, moderate, severe. To address the frequent uncertainty surrounding certain symptoms in clinical practice, we have added the category “questionable/subthreshold”. Additionally, an overall severity rating is recommended using the Adjusted Clinical Global Impression Scale [27] (0–4 rating, where 0 = not present, 1 = questionable, 2 = mild, 3 = moderate, and 4 = severe)**.** The AMDP system is operationalized, well-validated and manualized. It is available in multiple languages [25], maintained by an AMDP society that offers training sessions, and has been in used for over 50 years [26]. Its foundation lies in the psychopathological descriptions of Jaspers [28] and Schneider [29] which were based on clinical observations. However, it may eventually be replaced by a cross-diagnostic instrument developed according to modern standards [30]. For a more detailed description of symptoms, a second-level analysis can be presented (eFigure 1). Once validated, the Transdiagnostic Global Impression Scale may be an option, as well [31].

In the example case, the patient primarily experiences negative symptoms and a cognitive deficit**,** the latter being a common predictor of poor functioning across many psychiatric disorders [32]. Additionally, the patient suffers from depression and anxiety, as well as some positive symptoms in the form of hallucinations and delusions. The patient also exhibits motor symptoms, which may be attributed to haloperidol medication (Fig. 2a).

It should be noted that if such a spidernet plot were implemented in a smartphone or computer application, different ways of sorting the symptoms could be provided with one click. Moreover, the change of symptoms over time could be displayed.

Further Clinical Characterisation (Fig. 2a, right): The patient is characterized by the following factors, which contribute to the mental disorder and form problems to be addressed. Their selection was guided by publications on depression [33] and primary psychotic disorders [34] (see also [9]):

- Even in the absence of a diagnosed personality disorder, understanding a patient’s personality style is important. It can guide interactions with the patient and ensure that psychotherapeutic treatments are appropriately tailored to their needs [9].
- Intelligence, measured by a brief IQ test such as the Kaufman Brief Intelligence Test [35] or the Wechsler Abbreviated Scale of Intelligence (WASI) [36] can be important in two ways. One is to screen for intellectual disability. We have added this suggestion to Fig. 2a. In contrast, high IQ is a resource which has been shown to be a predictor of good outcome to psychotherapy [37] Therefore, we added high IQ of 120 to Fig. 2b as a personal strength of the theoretical patient.
- Physical diseases can either directly impact brain function (e.g. HIV, stroke) or be a psychological burden. Moreover, psychiatrists must take care of relevant comorbid physical illnesses, such as obesity resulting from psychotropic medication.
- Understanding a patient’s psychiatric history and staging are essential for prognosis.
- Staging (0–4 according to Scott et al. 2024) [38]. The primary goal of staging aims is preventive treatment. We need to intervene as early as possible to avoid chronification. Moreover, treatment should be tailored to the specific stage of the disorder, with earlier stages typically requiring less intensive approaches [39].
- While family history is not a modifiable risk factor that can be directly treated, it provides valuable insight into the biological components and potential learning history underlying a mental health issue.
- Substance and alcohol use disorders can both cause and perpetuate mental problems. Identifying and addressing such problems is essential for achieving optimal outcomes.
- Earlier and recent life events, family or partner conflicts are potential causes of a mental problem which need to be addressed.
- The same applies to *self-stigma* and the stage of personal recovery, where peer-support may be especially valuable [40].
- Social problems such as loneliness, unemployment, poverty and discrimination are significant causative factors [41] and must be addressed, often with the help of social workers and peer-support workers. Conversely, the absence of such problems can act as a protective factor and as a resource.
- Lack of concordance in medication use is frequent and needs to be recognized, as it cannot be effectively addressed otherwise.

This list contains characteristics that are important for most disorders. Moreover, *problem-specific characteristics* such as dysfunctional cognitive schema in depression [33], duration of untreated psychosis in first-episode psychosis, which is important for prognosis [34], specific, e.g. genetic, tests for patients with intellectual disability, or speech difficulties in neurodevelopmental disorders can be added.

In the example obesity, elevated liver enzymes and cannabis use must certainly be considered in treatment plans.

Resources (Fig. 2b): Finally, in mental health care it is important to not only focus on deficits, but to also consider the patient’s resources and resilience related factors [42]. To this end, Fig. 2b presents another spiderweb plot outlining the major personal resources as defined by Priebe et al. [42].

The example patient’s resources include strong ties with family and friends, access to professional care, financial means and individual strengths such as good communication skills.

- 2. Goal-adapted, prioritized and hierarchised problem list (Fig. 3, left)

The problems identified in the previous section need to be *hierarchised*. For example, not all problems can be addressed in an inpatient stay. Incorporating the patient’s goals through a shared-decision-making process [43] is a key addition to Weed’s original proposal. One would also delve into the persons’ values and areas where they find meaning in life. In cases such as pneumonia, curing the illness is naturally aligned with the patient’s goal. In mental health care, the situation can be different. For example, some patients with chronic schizophrenia may not prioritize completely eliminating hallucinations if it involves significant side effects. Instead, they might prefer to reduce symptoms to a level that enables them to engage in social activities. The concept of *“personal recovery”* is central to this approach. While societal recovery – such as starting a family, gaining a university degree or having a full-time job is often not realistic. If imposed on patients they may overextend themselves and experience frustration. *Personal recovery* means to re-gain well-being and meaning in life by striving for one’s individual goals [40].

In Figs. 2a and 3, positive symptoms are prioritized, as other symptoms—such as anxiety, depression, negative symptoms, and even cognitive deficits (which may result from formal thought disorder)—are often secondary to positive symptoms and may improve in parallel when the former are resolved. Obesity and elevated liver enzymes represent significant physical health concerns that require immediate attention, particularly in the context of selecting antipsychotic medication. Addressing cannabis use is also critical, as its continued use may hinder improvement in psychotic symptoms. Cognitive training and vocational planning are typically more appropriate toward the end of an inpatient stay.

Figure 3 includes a broad range of psychotherapeutic interventions to emphasize that the treatment of psychosis should not rely solely on antipsychotic medication. Numerous non-pharmacological treatment options are available; however, not all interventions may be accessible or feasible to implement simultaneously. Therefore, when developing a treatment plan, it is advisable to begin by brainstorming potential interventions, which can then be selected and tailored based on the individual needs of the patient.

- 3. Formulating treatment plans (Fig. 3 right)

According to Weed, it is crucial to collaboratively develop a treatment plan for each identified problem. Figure 3 presents treatment plans for the example patient, along with the therapist responsible for each task. Its ability to foster collaborative and interdisciplinary care is a major advantage of POMR. Without jointly formulated treatment plans, team members and the various institutions involved in care may sometimes work independently. This fragmentation partly arises from the structure of source-oriented medical records, where information from each party is stored separately, limiting coordination involving all parties in the planning process could enhance collaboration and improve team satisfaction.

A problem-oriented treatment plan in psychiatry should be co-created with the patients, enabling them to actively participate in their care. Central to this approach is respecting the patient’s voice and preferences, supporting their autonomy while addressing the biological, psychological, social, and existential dimensions of their struggles. Mental suffering is a complex, multi-dimensional phenomenon, and this requires adopting a pluralistic approach that acknowledges the various perspectives through which mental health and illness can be understood. A comprehensive treatment plan should not only focus on symptom reduction but also promote personal recovery by helping individuals find meaning and value in their lives, even in the presence of ongoing symptoms or disabilities. The plan should aim to support the development of resilience and coping skills, tailored to the individual’s strengths and life context. This includes fostering social skills, emotional regulation, and self-management strategies, empowering the patient to navigate challenges and setbacks in a way that aligns with their values and fosters personal growth.

The treatment plan should be integrated within a “mental health ecosystem” [44] which refers to a network of collaborative partners in primary care, mental health, social care, recovery colleges, informal support, complementary care, and digital communities. This ecosystem approach provides a comprehensive context for addressing problems that go beyond the traditional medical model. Patients are encouraged to actively engage in their recovery process by navigating this ecosystem, giving them the opportunity to explore various treatment modalities that align with their needs, preferences, and personal recovery goals. The plan would offer flexibility, allowing the patient to choose where to begin within the ecosystem—be it through psychotherapy, social support, recovery academies, or even online self-help communities.

Integrating these biopsychosocio-existential and ecosystem principles can transform the problem-oriented treatment plan in psychiatry into a dynamic, patient-centred process. This approach shifts the focus from merely symptom reduction to also fostering a life of meaning and well-being, recognizing each individual’s unique journey within the broader social and existential context.

- 4. Progress notes (Fig. 4)

The final step of POMR, progress notes should follow Weed’s general approach, with one exception: the “objective (O)” component in the SOAP format is by nature less the focus in mental health care. However, standardized and validated methods to access psychopathology, resources and deficits form the “O” in the psychiatric setting within the SOAP scheme.

In some countries, paper health records are still in use, but in the medium-term, electronic health record systems will become the standard. These electronic systems must be designed in a way that fully supports the implementation of the problem-based approach.

- 5. Knowledge Tools (Fig. 1, middle)

Finally, it is important to note that Weed advocated for computerized tools. Patient characteristics should be linked to a knowledge database, and potential diagnoses should be identified using computer software. He rightly argued that clinicians cannot know all medical knowledge, process it and apply it to individual patients. As a result, clinicians often rely on a mixture of knowledge, experience, “clinical wisdom”, “intuition” and “guesswork” [11]. This insight remains crucial and computer-assisted medicine represents the future. In mental health care, this is less straightforward as diagnoses are primarily based on psychopathological signs rather than biological tests. Nevertheless, Figs. 2 and 3 already represent a basic electronic tool, and treatment guidelines could be implemented in electronic health records. Such integration would encourage more frequent use of these guidelines, improving decision-making in clinical practice.

## Conclusion

Psychiatric problems are syndromes, not distinct diagnostic entities with clear biological pathologies. They are multi-dimensional, encompassing biological, psychological, and social aspects. This complexity makes them particularly suited for a problem-oriented approach, which emphasizes symptoms and contextual characterization, coupled with goal-setting and resource-oriented strategies, rather than solely relying on ICD/DSM diagnoses. This approach systematically and holistically embraces patients, emphasizing patient related goals and personal recovery over societal expectations. We refer to this approach as the Problem–Resources–Goals Oriented framework (PRoGO), reflecting the necessary adaptations for mental health care. The aim is that such an approach will enhance treatment outcomes, as well as patient and team satisfaction. Trials to assess its feasibility, effectiveness, and utility in psychiatry are warranted. Before these can be conducted, many issues such as how POMR/PRoGO can be integrated into the current health record systems, and how it can be fitted into the clinical workflows need to be considered and piloted.

## Supplementary Information

Below is the link to the electronic supplementary material.

Supplementary file1 (DOCX 49 KB)
