# Supplementary material for: Enhancing mental health care: a problem, resources and goals oriented multidimensional framework (PRoGO)
Source: Eur Arch Psychiatry Clin Neurosci. 2025 Jul 1;276(1):51–61. doi: 10.1007/s00406-025-02045-5 (PMC12904919; doi:10.1007/s00406-025-02045-5)
Supplement: Supplementary file 1 — Supplementary file1 (DOCX 49 KB) [file 406_2025_2045_MOESM1_ESM.docx]

**eAppendix 1: Narrative Review of Studies on the Problem-Oriented Medical Records Approach in Psychiatry**

**Search Strategy**

We searched Pubmed with the term ("Lawrence Weed" OR "Problem-oriented" OR POMR OR SOAP OR POC) AND ("Mental Health" OR Psych* OR Neuropsychiatry OR Depress* OR Anxiety OR Bipolar OR Schizo* OR "Personality disorder*" OR "Mood Disorder*" OR "Eating Disorder*" OR "Post-traumatic" OR PTSD OR "Obsessive-compulsive" OR OCD OR Addiction* OR Neuroscience OR "Cognitive-Behavioral" OR "Cognitive Behavioral" OR Antidepressant* OR Antipsychotic* OR "Mood Stabilizer*" OR Suicide OR Mindfulness) (last search 26/09/2024).

There were 1805 hits of which 23 articles were relevant.

We categorized the articles around the themes (1) “Presentation of the Problem-Oriented Approach”, (2) “Technical Aspects of the P-O Approach”, (3) “Evaluation of the Effectiveness of the P-O Approach” and (4) “Interventions to Increase the Application of the P-O Approach”.

In the following text we first present a narrative summary of the single studies. Subsequently the most important information derived from these articles is summarized in tables.

**1. Presentation of the Problem-Oriented Approach**

**Chopra (1980)** criticized the inefficiency of traditional medical records in psychiatry. He introduced the Problem-Oriented Record (POR) as a systematic method comprising a Database, Problem List, Treatment Plan, and Progress Notes. The POR improves problem definition, treatment documentation, and systematic evaluation, enhancing logical thinking and communication. However, potential limitations include the fragmentation of problems and the necessity to incorporate patient resources.

**Hayes-Roth (1972)** emphasized the crucial role of organized medical records in psychiatric care. He introduced the POMR as a solution to disorganized information systems, detailing components like the Data Base, Problem List, Plans, and Follow-up. Hayes-Roth proposed transitioning to computerized systems and standardizing data collection to improve information organization, research capabilities, and interdisciplinary communication. The challenges highlighted included the necessity for philosophical and operational changes in the psychiatric system.

**Lang (1985)** presented a structured, goal-oriented format to enhance the engagement of activity therapy disciplines in multidisciplinary treatment planning. The new format defined specific and measurable goals, increased accountability, reduced documentation time, and improved staff education. Patient participation was also enhanced, as patients better understood their goals and the steps needed to achieve them. Implementation challenges included staff resistance and overcoming perceptions that written documentation could lead to criticism.

**2. Technical Aspects of the Problem-Oriented Approach**

**Fowler (1975)** discussed the POMR's main components and highlighted challenges specific to psychiatry in defining problems. He emphasized the need for operational definitions of "problems" and the importance of separating data from assessments. Fowler discussed "lumping" versus "splitting" problems, advocating for criteria like covariance and equivalence to enhance problem formulation and clinical communication.

**Fahrner (1977)** identified shortcomings in the traditional record system at Ypsilanti State Hospital. Implementing Weed's POMR revealed issues like excessively long problem lists and tracking difficulties. Modifications such as developing a Processed-Problem List and a Problem Card helped manage major and minor issues more effectively, leading to improved clinical efficiency. This adaptation made treatment monitoring easier and reduced record clutter.

**Gifford (1979)** introduced the CHARTS system, a computerized patient record system with features like planned event capabilities and a master problem list. Advantages included reduced errors, efficient documentation, and enhanced research capabilities. Challenges involved initial data entry and gaining staff acceptance, which was achieved by involving staff in the system's design.

**Haber (1978)** adapted Weed's POMR to psychiatric contexts, outlining advantages like meaningful context for each problem, improved care continuity, and an organized format for medical education. Challenges included precise problem definitions, specific treatment documentation, and staff acceptance. The article underscored the nursing staff's role in interdisciplinary collaboration.

**Hammond (1984)** presented the Treatment Planner, a computerized psychiatric treatment planning system using the MUMPS language. Features included user interactivity and automatic generation of treatment plans. The system improved documentation efficiency and data retrieval. Challenges like data entry and clinician resistance were mitigated through staff involvement and system customization.

**Harvey (1974)** addressed the need for precise operational definitions of problems in psychiatry, moving beyond broad diagnostic labels. He recommended using the SOAP format for progress notes and highlighted the importance of integrating nursing activities. Challenges included training needs and the potential risk of fragmenting patient care.

**Meldman (1977)** described a computer-assisted, goal-oriented progress note system based on Goal Attainment Scaling. The system allowed flexible data input and automatically generated structured progress notes. It improved documentation completeness, had high clinician acceptance, and enhanced clinical benefits through structured information organization.

**3. Evaluation of the Effectiveness of the Problem-Oriented Approach**

**Summary:**

Research in this category assesses the POMR's validity, reliability, and impact on therapeutic outcomes in psychiatry.

**Allen (1980)** introduced the POMR system to enhance therapeutic outcome evaluations in mental health. By integrating treatment plans with graphical records, the system effectively monitored changes in patient issues. The study demonstrated significant validity by correlating the POMR with independent measures like the Taylor Manifest Anxiety Scale, concluding that it is a valid and efficient method for documenting and evaluating outcomes.

**Biron (1977)** identified significant deficiencies in traditional medical records within a psychiatric center. By developing a POMR system with ten sections and a concise module for patient assessment and treatment planning, the institution improved documentation quality. Mandatory training sessions corrected common record-keeping errors, demonstrating that the POMR serves as both a documentation tool and an educational resource for clinical thinking.

**Draper (1975)** described the implementation of the POMR at the Virginia Treatment Center for Children. Motivated by inefficiencies and recommendations from the Joint Commission, the center adapted the POMR to suit child psychiatry's unique needs. The new system led to improved clinical efficiency, better educational tools for students, and clearer documentation of patient progress, receiving positive acceptance from the staff.

**Gilandas (1972)** reported on the adoption of the POMR at the North Dakota State Hospital. The approach included a Database, Problem List, Treatment Plan, and SOAP-formatted Progress Notes. Benefits observed were easier information retrieval, better treatment management, and enhanced documentation consistency. Challenges involved the need for precise problem definitions and adaptation to diverse theoretical orientations. The approach also officially recognized nurses' roles in defining and managing problems, promoting interdisciplinary collaboration.

**Novello (1973)** stressed the importance of accurate documentation in psychiatric practices. Implementing the POR in a psychiatric unit led to improved treatment planning, structured documentation, and facilitated multidisciplinary team involvement. Challenges included formulating problems in objective and measurable terms and managing emerging information. Involving patients in defining problems and goals enhanced communication and therapeutic effectiveness.

**Longabaugh (1983)** conducted a similar study, reinforcing the findings that both problems and diagnoses are essential for effective treatment planning. Specific problems predicted interventions independently of diagnoses, indicating that a problem-focused approach captures clinical nuances that diagnoses alone may miss.

**Scales (1975)** discussed the POMR's implementation at Fairfield Hills Hospital to meet Medicare documentation requirements and improve treatment coordination. The system's components included a color-coded organization for the Database, Problem List, Treatment Plans, and Progress Notes. Challenges faced were staff training and initial resistance due to increased workload. Benefits realized were improved organization of medical records, enhanced interdisciplinary collaboration, and better audit capabilities.

**Williams (1974)** introduced the POMR on a psychiatric inpatient unit to address disorganized and incomplete narrative records. The implementation involved staff training and developing standardized forms, leading to better problem identification, treatment planning, and interdisciplinary collaboration. Challenges included staff resistance and the need for additional training to improve clinical skills. The POMR facilitated audits and enhanced clinical learning through continuous documentation.

**Webb (1980)** investigated the POMR system's reliability in evaluating therapeutic outcomes through inter-rater agreement. The study found moderate reliability in identifying main problems but lower reliability in assessing severity. It highlighted the need for improved training to enhance reliability in severity assessments.

**4. Evaluation of Interventions to Increase the Application of the P-O Approach**

**Bakel (2014)** emphasized the importance of problem lists in medical records. The study implemented a quality improvement project to increase problem list usage in inpatient hospitals. Through educational interventions, procedural changes, and performance feedback, significant increases were achieved. Challenges remained in measuring the accuracy of problem lists and generalizing results.

**Gaviria (1976)** conducted an 18-month study implementing the POMR in eight psychiatric teams. The process involved standardized training and supervision. Results varied, with some teams fully adopting the approach and others struggling due to factors like leadership competence and team management. The study concluded that successful adoption requires procedural changes, philosophical shifts, and positive staff attitudes.

**Liston (1976)** surveyed community mental health centers associated with university residency programs. The survey revealed significant interest in the POMR, with perceived benefits like improved organization and supervision facilitation. Challenges included resistance from medical staff and adaptation difficulties, especially in psychodynamically oriented centers.

**Vickar (1976)** similarly evaluated the POMR's use in community mental health centers. Findings mirrored Liston's study, highlighting staff resistance, particularly from physicians, due to workload concerns. The study emphasized the need for training and support to overcome implementation challenges.

1. **Presentation of the Problem-Oriented Approach**

| **Reference** | **Title** | **Subject** | **Key Points** |
| --- | --- | --- | --- |
| Chopra_1980[1] | The Problem-Oriented Approach in Psychiatry | Presentation of the Problem-Oriented Approach | - Criticism of the traditional method of managing medical records, highlighting issues of efficiency and organization. - Introduction of the Problem-Oriented Record (P.O.R.) as a systematic method for coding medical data. - Description of the four sections of the P.O.R.: Database, Problem List, Treatment Plan, Progress Notes. - Comparison between traditional medical records and P.O.R., highlighting the advantages of P.O.R. in terms of problem definition, treatment documentation, and systematic evaluation. - Discussion on the benefits of P.O.R., including improvements in logical thinking, efficiency in record review, better communication, and support for clinical research. - Critical evaluation of the potential limitations of P.O.R., such as the fragmentation of problems and the need to include patient resources. |
| Hayes-Roth_1972[2] | The Problem-Oriented Medical Record and Psychiatry | Presentation of the Problem-Oriented Approach | - Description of the psychiatric context: The psychiatric healthcare system is described as an evolving social system that requires an effective information system to improve. - Importance of medical records: The medical record is identified as a crucial component for patient care, but currently disorganized and not very useful for institutional feedback. - Introduction of the P-O Approach: Lawrence Weed’s problem-oriented system is presented as a solution to organize information in a logical and useful way. - **Components of the System:** 1. Data Base: Collection of essential information (mental state examination, psychological assessments, social history, etc.). 2. Problem List: Numbered and categorized list of the patient’s problems. 3. Plans: Specific treatment plans for each problem. 4. Follow-up: Progress notes related to identified problems. - **Proposal for Improvement:** Suggests a transition to a computerized system and more efficient data collection through standardized questions. - **Expected Benefits:** Better organization of information, facilitation of research and audits, and improvement of interdisciplinary communication. - **Challenges:** Need for philosophical and operational changes to adapt the psychiatric system to the P-O Approach. |
| Lang_1985[3] | The Multidisciplinary Treatment Plan: A Format for Enhancing Activity Therapy Department Involvement | Presentation of the Problem-Oriented Approach | - Introduction of a Structured Format: Presents a structured and goal-oriented format to enhance the engagement of activity therapy disciplines in the multidisciplinary treatment planning process. - **Benefits of the Format:** - Specific and Measurable Goals: Definition of clear and achievable functional goals. - Increased Accountability: Enhanced accountability in treatment planning and delivery. - Reduction in Documentation Time: Optimization of the documentation process. - Improvement in Staff Education: Greater understanding of the role and function of activity therapy. - Patient Participation: Patients better understand their goals and the steps needed to achieve them, increasing participation in the therapeutic process. - **Challenges in Implementation:** - Staff Resistance: Some staff members may prefer verbal communication or oppose separating the treatment plan from progress notes. - Acceptance of the Format: Need to overcome the perception that written documentation might lead to criticism if patients do not achieve the defined goals. - **Practical Examples:** Describes how a new treatment plan format was developed and implemented, involving the leaders of activity therapy disciplines to define goals, methods, and progress indicators. - **Expected Outcomes:** Improved interdisciplinary communication, increased staff accountability, and clearer, more focused documentation on patient issues and goals. |

**2. Technical Aspects of the Problem-Oriented Approach**

| **Reference** | **Title** | **Subject** | **Key Points** |
| --- | --- | --- | --- |
| Fahrner_1977[4] | Record-Keeping in a State Hospital: A Modification of the Weed System | The Technical Aspects of the Problem-Oriented Approach | - Identification of deficiencies in the traditional medical record system at Ypsilanti State Hospital. - Implementation of Weed’s POMR with four main components: Database, Problem List, Treatment Plan, Progress Notes. - Issues encountered during the pilot: excessively long problem lists and difficulties in tracking specific treatments. - Modifications made: development of Processed-Problem List, Problem Card, and Unprocessed-Problem List to better manage major and minor issues. - Benefits of the modifications: easier monitoring of treatments, reduced clutter in records, and improved clinical efficiency. - Widespread adoption of the modified system throughout the hospital, with staff training and positive feedback. |
| Fowler_1975[5] | The Problem-Oriented Record. Problem Definition | The Technical Aspects of the Problem-Oriented Approach | - Detailed description of the Problem-Oriented Medical Record (POMR) and its main components. - Specific challenges in defining problems in psychiatry compared to other medical disciplines. - Operational definition of “problem” as a dysfunction perceived by clinical staff that affects the patient. - Importance of separating data from assessments to maintain clarity in documentation. - Discussion on the criteria of “lumping” (grouping) vs. “splitting” (dividing) problems, with theoretical criteria such as covariance, equivalence, and response to third variables. - Value of the POMR in improving problem formulation, data assessment, and clinical communication, increasing the precision and effectiveness of psychiatric treatment. |
| Gifford_1979[6] | An Integrated System for Computerized Patient Records | The Technical Aspects of the Problem-Oriented Approach | - Description of the CHARTS system (Community Health Automated Record and Treatment System) developed at the Heart of Texas Region Mental Health-Mental Retardation Center. **Innovative Features:** - Planned event capability: automatic entry and reminders for planned events and activities. - Individual direct service record: automatically generated forms for each direct service event, with problem severity ratings. - Client level of functioning scale: a scale that evaluates the overall functioning of the client in nine areas. - Master problem list: centralized and coded list of problems, facilitating data collection and consistency in documentation. **Advantages:** - Error reduction: significant decrease in the error rate in clinical data. - Documentation efficiency: creation of clear and concise treatment plans, easily accessible and comparable. - Facilitation of research and audit: ability to quickly extract and analyze clinical data for research and quality control purposes. **Challenges Faced:** - Initial data entry: need to avoid excessive entry of historical data. - Staff acceptance: initial resistance from some clinicians, overcome by involving staff in the system design. **Conclusions:** - The CHARTS system has improved the quality and efficiency of psychiatric medical records. - Future developments: extension of the system to other clinical documents and integration with rule-based decision support. |
| Haber_1978[7] | The Problem-Oriented Record in Psychiatry | Technical Aspects of the Problem-Oriented Approach | **Introduction to the P-O Approach:** Adaptation and implementation of Weed’s P-O Approach in psychiatric contexts. **Advantages of the P-O Approach:** - **Meaningful context:** Each problem is placed in a context that facilitates understanding and treatment. - **Continuity of care:** Improved tracking and documentation of the progression of the patient’s problems. - **Scientifically acceptable record:** Clear and structured documentation that facilitates audits and evaluations. - **Organized format for medical education:** The structure of the record supports the teaching and training of healthcare professionals. - **Meaningful audits:** Ability to assess the effectiveness and efficiency of care through detailed documentation. **Challenges in Implementation:** - **Precise definition of problems:** Need for operational and behavioral descriptions of problems to avoid vagueness and ambiguity. - **Specific documentation of treatments:** Clear and precise detailing of therapeutic methodologies applied to each problem. - **Staff acceptance:** Need for training and philosophical change to effectively adopt the system. - **Contribution of nursing staff:** Integration and documentation of nursing activities in the P-O Approach. **Conclusions:** - The P-O Approach significantly improves the quality of psychiatric documentation. - **Recommendations:** Promote adequate training and operational definition of problems to maximize the system’s effectiveness. |
| Hammond_1984[8] | A Computerized Psychiatric Treatment Planning System | The Technical Aspects of the Problem-Oriented Approach | - **Description of the Treatment Planner:** A computer-assisted system developed at the Jerry L. Pettis Memorial Veterans Hospital. **System Features:** - Use of File Manager and MUMPS language: Facilitates the definition, manipulation, and modification of informational files without requiring advanced programming skills. - Interactivity: Dialogue between user and computer to simplify the selection and description of issues. - Automatic generation of treatment plans: Production of structured paragraphs based on user choices. - Categorization of issues: Creation of coded problem lists to facilitate data analysis and research. **Advantages:** - Efficiency in documentation: Reduction in the time required for writing and managing treatment plans. - Ease of data retrieval and analysis: Ability to quickly extract and analyze clinical data for research and audit purposes. - Staff acceptance: Involvement of staff in the system design has increased acceptance and satisfaction. **Challenges Faced:** - Data entry: Need to avoid excessive entry of historical data. - Initial resistance: Some clinicians showed resistance to adopting the system, which was overcome through involvement and customization. **Conclusions:** - The Treatment Planner has improved the quality and efficiency of psychiatric medical records. - Future developments: Extension of the system to other clinical documents and integration with rule-based decision support systems. |
| Harvey_1974[9] | Toward a More Efficient Use of the Problem-Oriented Record in Psychiatry | Technical Aspects of the Problem-Oriented Approach | - **Specific Challenges of the P-O Approach in Psychiatry:** Difference between broad diagnostic labeling and the need for precise operational definitions. - **Problems with Traditional Labels:** Diagnoses like “paranoid schizophrenia” do not reflect specific behaviors and require detailed descriptions. - **Operational Definition of Problems:** Importance of describing problems in terms of observable and specific behaviors. - **SOAP Format:** Use of the SOAP format (Subjective, Objective, Assessment, Plan) to structure progress notes. - **Contribution of Nursing Staff:** Recognition and documentation of nursing activities in the P-O Approach. - **Advantages of the P-O Approach:** - Greater accuracy and specificity in the documentation of problems and treatments. - Facilitation of audit and treatment evaluation. - Improvement of interdisciplinary communication. - **Challenges:** - Need for training: Requires a philosophical and practical change in documentation. - Risk of fragmentation: Criticisms from approaches like Gestalt that see the system as fragmenting the patient. - **Conclusions:** - The P-O Approach requires precise operational definitions to be effective in psychiatry. - **Recommendations:** Promote detailed and behavioral documentation to maximize the benefits of the system. |
| Meldman_1977[10] | A Computer-Assisted, Goal-Oriented Psychiatric Progress Note System | The Technical Aspects of the Problem-Oriented Approach | - **Introduction of Computerization:** Development of a computer-assisted psychiatric progress note system oriented towards goals. **Computerization Requirements:** - Conceptual model based on Goal Attainment Scaling to align the system with clinical practices. - Flexibility in data input: Allows for free input of narrative text in addition to structured selections. - Educational function: The system acts as an extension of clinical memory and reduces errors of omission. - Clinical and administrative utility: Progress notes are useful both for clinicians in patient treatment and for administrative and audit reviews. **System Design:** - Frame Library: Hierarchical organization of data options through frames (Directory Frames, Progress Note Frames, Assistance Frame). - System Output: Generation of structured progress notes and historical database for retrospective research. **Results:** - Improvement in documentation: Greater completeness and timeliness in the entry of progress notes. - Acceptance by clinicians: High acceptance due to flexibility and reduced time required for documentation. - Clinical advantages: Structured organization of information that facilitates the review and monitoring of patient progress. **Conclusions:** - System effectiveness: The computerized system has improved the quality and efficiency of clinical documentation in psychiatry. - Conceptual resonance: The success of the system depends on its consistency with the goal-oriented conceptual model of the therapeutic process. |

**3. Evaluation of the Effectiveness of the Problem-Oriented Approach**

| **Reference** | **Title** | | | **Subject** | **Key Points** | |
| --- | --- | --- | --- | --- | --- | --- |
| Allen_1980[11] | Validity of Problem-Oriented Record System for Evaluating Treatment Outcome | | | Evaluation of the effectiveness of the Problem-Oriented Approach | - Introduction of the Problem-Oriented Record System to optimize the evaluation of therapeutic outcomes in mental health settings. - Integration of the treatment plan and a graphic record to monitor changes in identified issues. - Study on the concurrent validity of the system using correlations with independent measures (e.g., Taylor Manifest Anxiety Scale). - Results indicate significant validity of the system in measuring changes in patients’ clinical status. - Conclusion: The P-O system is a valid and cost-effective method for documenting and evaluating therapeutic outcomes without overburdening the staff. | |
| Biron_1977[12] | The Problem-Oriented Medical Record as a Training Tool for Staff | | | Evaluation of the effectiveness of the Problem-Oriented Approach | - Identification of deficiencies in traditional medical record systems in a psychiatric center. - Development of a POMR system with ten sections and a four-page module for patient assessment and treatment planning. - Implementation of mandatory two-hour training sessions for all staff, followed by advanced 20-hour training. - Identification and correction of common errors in record-keeping, improving the quality of medical records. - Conclusion: The POMR not only enhances documentation but also serves as an effective educational tool for clinical thinking and treatment planning. | |
| Draper_1975[13] | Introduction of the Problem-Oriented Record into a Child Psychiatric Hospital | | | Evaluation of the effectiveness of the Problem-Oriented Approach | - Implementation of the POMR at the Virginia Treatment Center for Children, a psychiatric institution for children. - Reasons for the change: inefficiencies in the traditional record-keeping system and recommendations from the Joint Commission. - Steps for implementation: literature review, visit to another hospital using POMR, staff training, and pilot cases. - Specific adaptations for child psychiatry, such as managing multiple and flexible issues. - Observed benefits: improved clinical efficiency, better educational tool for students, and greater clarity in documenting patient progress. - Positive acceptance by staff and widespread adoption of the system throughout the hospital. | |
| Gilandas_1972[14] | The Problem-Oriented Record in a Psychiatric Hospital | | | Evaluation of the effectiveness of the Problem-Oriented Approach | - Introduction of the P-O Approach at the North Dakota State Hospital, a psychiatric institution with three regional units. **Components of the P-O Approach:** 1. Database: Collection of essential information such as mental status examination, physical reports, psychological assessments, social history, etc. 2. Problem List: A numbered and titled list of the patient’s problems, continuously updated. 3. Treatment Plan: Treatment plan for each identified problem, with responsibilities assigned to the appropriate staff. 4. Progress Notes: Progress notes related to problem numbers, structured in SOAP format (Subjective, Objective, Assessment, Plan). **Preliminary Impressions:** - Benefits: Easier information retrieval, better management of treatments, greater consistency in documentation. - Challenges: Need for precision in problem definition, adaptation to the diverse theoretical orientations of the clinical team. **Contribution of Nursing Staff:** Official recognition of nurses’ role in defining and managing problems, enhancing interdisciplinary collaboration. **Conclusions:** - The P-O Approach has improved the efficiency and quality of psychiatric medical records. - Recommendations: Continue to promote precision in problem definition and interdisciplinary collaboration to maximize the benefits of the system. | |
| Longabaugh_1983[15] | Validation of a Problem-Focused Nomenclature | | | Evaluation of the effectiveness of the Problem-Oriented Approach | - **Research Objective:** To evaluate the utility of existing diagnostic nomenclatures (DSM-III and problem-oriented records) in supporting clinical decision-making in psychiatry. - **Methodology:** - Sample: Psychiatric patients undergoing treatment in a hospital, with data collected from problem-oriented medical records and DSM-III diagnoses. - Coding: Problems were classified into physiological, psychological, and social categories; treatments were separated into assessments and interventions. - Data Analysis: Multiple regressions were used to predict interventions based on diagnoses and problems. - **Results:** - Medical Interventions: Both problems and diagnoses explain significant variance in pharmacological interventions. - Psychological and Social Interventions: Problems are stronger predictors than diagnoses. - Combination of Problems and Diagnoses: Explains more variance than each alone, indicating that both provide useful and complementary information. - Specificity of Problems: Some problems predict specific interventions independently of diagnoses, suggesting that diagnoses alone do not capture all relevant clinical nuances. - **Conclusions:** - Integration of Problems and Diagnoses: Both are necessary and useful for effective treatment planning. - Limitations: A large portion of the variance in interventions remains unexplained, indicating the presence of other influencing factors. - Clinical Implications: There is a need for a nomenclature that integrates DSM-III diagnoses and clinical problems to maximize effectiveness in treatment planning. - Future Directions: Study the impact of the P-O Approach on patient health outcomes and assess whether integrating problems and diagnoses improves these outcomes. | |
| Novello_1973[16] | | The Problem-Oriented Record in Psychiatry | Presentation of the Problem-Oriented Approach | - **Importance of Accurate Documentation:** Emphasizes the importance of precise record-keeping in hospital psychiatric practices to improve patient care and facilitate professional learning. - **Introduction of the POR in a Psychiatric Unit:** - **Practical Implementation:** Describes the implementation of a Problem-Oriented Record (POR) system in a psychiatric unit with 28 patients. - **System Components:** Includes intake history, planning conference, problem list, progress notes, progress chart, flow sheet, progress conference, and discharge conference. - **Benefits of the POR:** - Improved Treatment Planning: A numbered list of problems that facilitates tracking and updating patient issues. - Structured Documentation: Progress notes linked to problem numbers to ensure consistency and traceability. - Multidisciplinary Team Involvement: Facilitates collaboration among various clinical disciplines through a common framework. - Support for Research and Evaluation: Provides structured data useful for outcome studies and evaluations of therapeutic effectiveness. - **Challenges and Solutions:** - Problem Formulation: Need to describe problems in objective and measurable terms, avoiding diagnostic ambiguities. - Management of Emerging Information: Use of flow sheets and progress charts to monitor advancements and identify new issues. - Patient Involvement: Include the patient in defining problems and goals to enhance communication and therapeutic effectiveness. - **Conclusions:** - Effectiveness of the POR: The POR is superior to the traditional system for clinical purposes but requires integrations to meet administrative and research needs. - Need for Objective Measurements: Implementation of goal attainment scaling to effectively measure therapeutic outcomes. - Future Prospects: Improve the POR to include post-treatment follow-up and more detailed evaluations of outcomes. | |  |
| Scales_1975[17] | | A Psychiatric POMR for Use by a Multidisciplinary Team | Evaluation of the effectiveness of the Problem-Oriented Approach | - **Introduction of POMR:** Implementation of the Problem-Oriented Medical Record (POMR) at Fairfield Hills Hospital, integrated with multidisciplinary teams. - **Motivations:** Addresses the need for consistent documentation to meet Medicare requirements and improve treatment coordination. - **Components of POMR:** Includes database, problem list, treatment plans, progress notes, with color-coding to facilitate organization. - **Challenges:** - Need to adapt POMR to the specific needs of the hospital. - Staff training. - Initial resistance due to the additional workload. - **Benefits:** - Improved organization of medical records. - Facilitation of interdisciplinary collaboration. - Enhanced documentation for audits and treatment evaluation. - **Conclusions:** - POMR has significantly improved care recording, team coordination, and audit capability, demonstrating its value in a multidisciplinary setting. | |  |
| Williams_1974[18] | | Introducing the Problem-Oriented Record on a Psychiatric Inpatient Unit | Presentation of the Problem-Oriented Approach | - **Motivations for the Introduction of the POMR:** Pressures for audit and review of the use of medical records, need to improve the organization and completeness of records. - **Unit Context:** Psychiatric unit with multidisciplinary staff, previously with disorganized and incomplete narrative records. - **Implementation of the POMR:** - **Planning and Training:** Creation of a committee to tailor the POMR to the unit’s needs, development of standardized forms. - **Components of the POMR:** Intake history, planning conference, problem list, progress notes, progress chart, flow sheet, progress conference, discharge conference. - **POMR Forms:** Specific forms for different aspects of treatment, maintained color-coded for disciplines, use of team meetings to complete the forms. - **Challenges:** - Staff resistance: Concerns about the additional workload and the quality of writing. - Improvement of clinical skills: Need for additional training to enhance interviewing and documentation skills. - **Benefits:** - Better organization: Quick identification of problems, improved treatment planning. - Interdisciplinary collaboration: Facilitates collaboration among different clinical disciplines. - Facilitation of audit: Improved ability to monitor and audit the quality of care and staff competencies. - Clinical learning: Enhancement of clinical skills through continuous documentation. - **Conclusions:** - The POMR significantly improved care documentation, team collaboration, planning, and audit capacity, representing an advantage over narrative records. | |  |
| Webb_1980[19] | Reliability of a Problem-Oriented Record System Approach to the Evaluation of Treatment Outcome | | | Evaluation of the effectiveness of the Problem-Oriented Approach | - **Purpose of the Research:** To investigate the reliability of the POMR system in evaluating therapeutic outcomes through rater agreement. - **Methodology:** - Sample: 32 outpatient clients, audio recordings of initial interviews. - Evaluation: Four therapists constructed POR for each client, selected the main problem, and assessed its severity. - Analysis: Percentage of agreement and Pearson correlation coefficients between raters for problems and severity. - **Results:** - Agreement on main problems: 63% average pairwise agreement, ranging from 60% to 68%. - Agreement on severity: 48.1% average, ranging from 26.3% to 71%. - Overall reliability: Reliability coefficient of .40, indicating moderate reliability. - **Conclusions:** - POR reliability: Provides moderate reliability in identifying main problems, but low reliability in assessing severity. - Implications: Need to improve training in using the system to increase the reliability of severity assessments. | |

**4. Evaluation of Interventions to Increase the Application of the P-O Approach**

| **Reference** | **Title** | **Subject** | **Key Points** |
| --- | --- | --- | --- |
| Bakel_2014[20] | A Quality Improvement Study to Improve Inpatient Problem List Use | Evaluation of interventions to increase the application of the P-O Approach. | - **Importance of Problem Lists:** Highlights their role in organizing medical records and supporting clinical decisions. - **Implementation of a Quality Improvement Project:** Aimed to increase the use of problem lists in inpatient hospitals above 80%. - **Interventions:** Included educational interventions, changes in documentation procedures, and performance feedback through periodic reports. - **Results:** Significant increase in the use of problem lists, with the medical team reaching a peak of 97% and the psychiatric unit 72%. - **Limitations:** Difficulty in measuring the accuracy of problem lists and the generalization of results to other contexts. |
| Gaviria_1976[21] | Changing to Problem-Oriented Methods. Implementation in Psychiatric Institutions | Evaluation of interventions to increase the application of the P-O Approach. | - **An 18-Month Study:** Implementation of the P-O Approach in eight psychiatric teams from different institutions. - **Operational Objectives:** Defining the recording system, quality control, and in-service training. - **Implementation Process:** Included standardized training, continuous supervision, and the use of pilot cases to test the system. - **Results:** - Variable success among the teams: two teams fully adopted the P-O Approach, three showed partial progress, and three faced significant difficulties or withdrew from the project. - Common inadequacies: Outdated problem lists, incomplete treatment plans, and inadequately structured progress notes. - Influence of team characteristics: Competent leadership and effective internal management facilitated the adoption of the system, while high turnover rates and poor organization hindered success. - **Conclusions:** - The adoption of the P-O Approach requires not only procedural changes but also philosophical modifications and positive attitudes from the staff. - **Recommendations:** An assessment of each unit’s readiness and direct involvement of clinical leadership are essential for successful implementation. |
| Liston_1976[22] | Use of Problem-Oriented Medical Record in Psychiatry: A Survey of University-Based Residency Training Programs | Evaluation of interventions to increase the application of the P-O Approach. | - **Purpose of the Research:** To investigate the adoption, acceptance, and perceived benefits of the P-O Approach in community mental health centers based on university residency training programs. **Methodology:** - **Sample:** 334 community mental health centers, with 158 responses received (47% response rate). **Results:** - **Adoption of the P-O Approach:** 24% of centers were already using the P-O Approach, 23% planned to implement it, 16% were uncertain, and 37% had not considered it. - **Perceived Benefits:** Improved organization, readability, and identification of problem areas. Facilitation of resident supervision and data collection for research. - **Perception of Clinical Interventions:** 64% of centers using the P-O Approach believed that patients benefited from a more consistent approach in documentation. - **Resistance from Medical Staff:** Physicians were less favorable towards adopting the P-O Approach compared to other team members, citing difficulties in adapting and resistance to change. - **Theoretical Orientation:** No significant difference in the adoption of the P-O Approach based on theoretical orientation, but psychodynamic centers had more difficulty adapting the system. - **Critiques:** Increased clerical workload, duplication of information, and inefficiency in the documentation system. **Conclusions:** - **Growing Interest:** There is significant interest among community mental health centers in adopting the P-O Approach. - **Challenges in Implementation:** Resistance from medical staff and the need for training and support to overcome difficulties in adapting the system. - **Future Prospects:** A potential increase in the adoption of the P-O Approach in response to the growing need for treatment evaluation and accountability, but it requires a philosophical and operational shift among clinicians. |
| Vickar_1976[23] | The Use of Problem-Oriented Mdical Records in Community Mental Health Centers | Evaluation of interventions to increase the application of the P-O Approach. | - **Research Objective:** Evaluate the adoption, acceptance, and perceived benefits of POMR in community mental health centers. - **Methodology:** Survey sent to community mental health centers, analysis of responses on the dissemination of POMR, benefits, and resistance. - **Results:** - **Adoption of POMR:** 24% of centers used it, 23% planned to implement it, 37% had not considered it. - **Perceived Benefits:** Improved organization, readability, identification of problem areas, facilitation of supervision and data collection. - **Staff Resistance:** Physicians showed more resistance compared to other team members, citing additional workload and difficulty in adaptation. - **Theoretical Orientation:** No significant difference based on theoretical orientation, but psychodynamic centers experienced more difficulties. - **Criticisms:** Increase in clerical workload, duplication of information, inefficiency in the documentation system. **Conclusions:** - Significant interest in the adoption of POMR, but challenges in implementation due to medical staff resistance and the need for training and support. - **Future Prospects:** Potential increase in the adoption of the P-O Approach in response to the growing need for treatment evaluation and accountability, but it requires a philosophical and operational shift among clinicians. |

**References**

1. Chopra, H.D., *The problem-oriented approach in psychiatry*

Indian J Psychiatry, 1980.

2. Hayes-Roth, F., R. Longabaugh, and R. Ryback, *The problem-oriented medical record and psychiatry.* Br J Psychiatry, 1972. **121**(560): p. 27-34.

3. Lang, E. and M. Mattson, *The Multidisciplinary Treatment Plan: A Format for Enhancing Activity Therapy Department Involvement.* Psychiatric Services, 1985. **36**(1): p. 62-68.

4. Fahrner, B.G., et al., *Record-keeping in a state hospital: a modification of the Weed system.* Hosp Community Psychiatry, 1977. **28**(12): p. 907-8.

5. Fowler, D.R. and R. Longabaugh, *The problem-oriented record. Problem definition.* Arch Gen Psychiatry, 1975. **32**(7): p. 831-4.

6. Gifford, S. and D. Maberry, *An integrated system for computerized patient records.* Hosp Community Psychiatry, 1979. **30**(8): p. 532-5.

7. Haber, J., *The problem-oriented record in psychiatry.* Issues Ment Health Nurs, 1978. **1**: p. 91-102.

8. Hammond, K.W. and T.H. Munnecke, *A computerized psychiatric treatment planning system.* Hosp Community Psychiatry, 1984. **35**(2): p. 160-3.

9. Harvey, R.T., et al., *Toward a More Efficient Use of the Problem-Oriented Record in Psychiatry.* Psychiatric Services, 1974. **25**(1): p. 42-43.

10. Meldman, M.J., et al., *A computer-assisted, goal-oriented psychiatric progress note system.* Am J Psychiatry, 1977. **134**(1): p. 38-41.

11. Allen, R.H., L.J. Webb, and R.S. Gold, *Validity of problem-oriented record system for evaluating treatment outcome.* Psychol Rep, 1980. **47**(1): p. 303-6.

12. Biron, R. and P. Goodman, *The problem-oriented medical record as a training tool for staff.* Hosp Community Psychiatry, 1977. **28**(12): p. 909-11.

13. Draper, W., *Introduction of the Problem-Oriented Record into a Child Psychiatric Hospital.* Journal of the American Academy of Child Psychiatry, 1975. **14**(1): p. 125-131.

14. Gilandas, A.J., *The problem-oriented record in a psychiatric hospital.* Hosp Community Psychiatry, 1972. **23**(11): p. 336-9.

15. Longabaugh, R., et al., *Validation of a problem-focused nomenclature.* Arch Gen Psychiatry, 1983. **40**(4): p. 453-61.

16. Novello, J.R., *The Problem-Oriented Record in Psychiatry.* The Journal of Nervous and Mental Disease, 1973. **156**(5): p. 349-353.

17. Scales, J.E. and M.S. Johnson, *A psychiatric POMR for use by a multidisciplinary team.* Hosp Community Psychiatry, 1975. **26**(6): p. 371-3.

18. Williams, D.H., et al., *Introducing the problem-oriented record on a psychiatric inpatient unit.* Hosp Community Psychiatry, 1974. **25**(1): p. 25-8.

19. Webb, L.J., et al., *Reliability of a problem-oriented record system approach to the evaluation of treatment outcome.* Psychol Rep, 1980. **46**(2): p. 452-4.

20. Bakel, L.A., et al., *A quality improvement study to improve inpatient problem list use.* Hosp Pediatr, 2014. **4**(4): p. 205-10.

21. Gaviria, B., J. Alvis, and N. Zarour, *Changing to problem-oriented methods. Implementation in psychiatric institutions.* J Nerv Ment Dis, 1976. **163**(2): p. 124-34.

22. Liston, E.H., *Use of problem-oriented medical record in psychiatry: a survey of university-based residency training programs.* Am J Psychiatry, 1976. **133**(6): p. 700-3.

23. Vickar, G.M., *The use of problem-oriented medical records in community mental health centers.* American Journal of Psychiatry, 1976. **133**(3): p. 340-341.
